# Modifizierte Masquelet-Technik zur Rekonstruktion kritischer Knochendefekte

**DOI:** 10.1007/s00113-024-01473-7

**Published:** 2024-08-14

**Authors:** Marc Hückstädt, Christian Fischer, Michael Mitin, Friederike Klauke, Steffen Langwald, Thomas Mendel, Philipp Kobbe, Sandra Schipper

**Affiliations:** 1 Klinik für Unfall- und Wiederherstellungschirurgie, BG Klinikum Bergmannstrost Halle gGmbH, Merseburger Straße 165, 06112 Halle, Deutschland; 2 Klinik für Unfall‑, Hand- und Wiederherstellungschirurgie, Universitätsklinikum Halle, Martin-Luther-Universität Halle-Wittenberg, Halle, Deutschland

## Einleitung

Die biologische Rekonstruktion knöcherner Defekte infolge von traumatischen Substanzverlusten, Infektionen oder Tumoren ist eines der anspruchsvollsten Gebiete in der muskuloskeletalen Wiederherstellungschirurgie. In aller Regel lassen sich kleinere Knochendefekte bis zu 3 cm relativ einfach durch Verkürzung oder primäre Spongiosaplastik erfolgreich behandeln [1, 2]. Größere Defekte erfordern hingegen den Einsatz aufwendiger rekonstruktiver Verfahren. Die Kallusdistraktion über einen Segmenttransport ist hierbei eine der am meisten verbreiteten Techniken, ist jedoch langwierig und komplikationsträchtig.

**Abb. 1:**
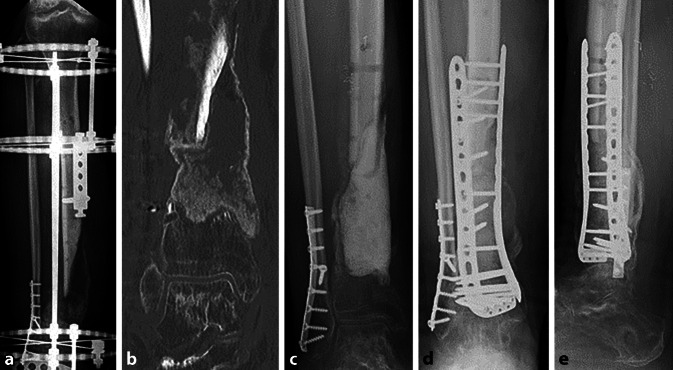
**a**,**b** Repositionsverlust und Sedimentationseffekt einer MT durch RIA und Ringfixateur; **c** Materialentfernung, Entfernung der Masquelet-Plastik und des avitalen Knochens, erneutes Einbringen eines Spacers ohne temporäre Osteosynthese; **d**,**e** MT durch zwei dekortizierte Femurköpfe und RIA, Doppelplattenosteosynthese, die Röntgenkontrolle nach zwei Monaten zeigt bereits eine Konsolidierung

Die Operationstechnik nach Masquelet (MT) ist ein zweizeitiges Rekonstruktionsverfahren zur Behandlung von Knochendefekten. Im ersten Schritt wird der Defekt durch einen Platzhalter aufgefüllt und durch eine Osteosynthese stabilisiert. Im Rahmen einer synovialen Fremdkörperreaktion wird eine Membran induziert, die u. a. durch eine gute Durchblutung und eine hohe Konzentration an Wachstumsfaktoren gekennzeichnet ist. Deshalb wird die Membran auch als Neoperiost bezeichnet. Im zweiten Schritt der MT werden der Platzhalter entfernt (meist nach 6 Wochen) und der Defekt unter Schonung der Membran mit Knochenersatzmaterial gefüllt [3–6]. Bei ausgedehnten Knochendefekten wird ein großes Volumen an Knochenersatzmaterial benötigt, weshalb sich die Gewinnung größerer Mengen autologen Knochens mit einem Reamer-Irrigator-Aspirator(RIA)-System etabliert hat. Auch wenn das RIA-Bone-Grafting biologisch hochpotentes Ersatzmaterial darstellt, kam es aufgrund der Sedimentation infolge der Schwerkraft häufig zu unbefriedigenden Ergebnissen (Abb. 1a, b; [7]).

Passgenaue industriell gefertigte 3D-Scaffolds können diesen Effekt verhindern, jedoch zeigen sie in der klinischen Anwendung einige Limitierungen (keine intraoperative Flexibilität, hohe Kosten, Aufklärung über individualisiertes Therapieverfahren etc.). Eine kostengünstige und intraoperativ auf den Defekt anpassbare Alternative bietet die Verwendung von allogenen Spenderfemurköpfen. Sie stellen in dieser alternativen Variante der MT das natürliche osteokonduktive Gerüst dar. Grundsätzlich können sowohl gefriergetrocknete (lyophilisierte) als auch thermodesinfizierte Femurköpfe verwendet werden, wobei wir ausschließlich thermodesinfizierte Köpfe verwenden, da diese sich aufgrund ihrer biomechanischen Eigenschaften besser verarbeiten lassen.

## Indikation

Die MT ist ein alternatives, innovatives Verfahren zum Segmenttransport (Kallusdistraktion), wobei in der Regel die Behandlungszeit kürzer und die Komplikationsrate geringer ist. Zudem können Knochendefekte jeder Lokalisation therapiert werden. Mit der MT können knöcherne Defekte nach Trauma, sanierter Infektion oder kurativer Tumorresektion rekonstruiert werden. Eine suffiziente Weichteildeckung ist dabei Voraussetzung [8–11].

Die Verfahrenswahl ist abhängig von der Compliance, den Nebenerkrankungen und der Lokalisation des Defektes. Metabolische Nebenerkrankungen wie z. B. ein Diabetes mellitus stellen bei guter medikamentöser Einstellung prinzipiell keine Kontraindikation dar.

Die MT ist vielfältig einsetzbar (u. a. für geriatrische Patienten) und kann nahezu bei jedem narkosefähigen Patienten durchgeführt werden. Lediglich Knochendefekte mit unmittelbarer Gelenkbeteiligung sind nicht oder lediglich unter Aufgabe des betreffenden Gelenkes im Sinne einer zusätzlichen Versteifung rekonstruierbar.

## Präoperative Planung und bildgebende Untersuchungen

Im Rahmen des individuellen Therapiekonzeptes werden in Vorbereitung auf das modifizierte Masquelet-Verfahren infektiologische oder onkologische Sanierungsoperationen, ggf. plastische Weichteildeckungen sowie additive medikamentöse Therapiemaßnahmen durchgeführt, um bestmögliche biologische Voraussetzungen für die Rekonstruktion zu schaffen. In den folgenden Ausführungen soll lediglich auf die verschiedenen chirurgischen Schritte der modifizierten MT eingegangen werden.

Die präoperative Vorbereitung beinhaltet regelhaft eine konventionelle Röntgenaufnahme der betroffenen Knochenregion in zwei Ebenen, abhängig von der Defektausdehnung mit Abbildung eines oder beider angrenzender Gelenke. Indikationsgerecht werden an den unteren Extremitäten zudem Ganzbeinaufnahmen oder Rotations-CT durchgeführt, um Beinlänge und -achse adäquat rekonstruieren zu können.

## Masquelet Schritt 1: Segmentresektion und Einbringen eines Platzhalters

**Abb. 2:**
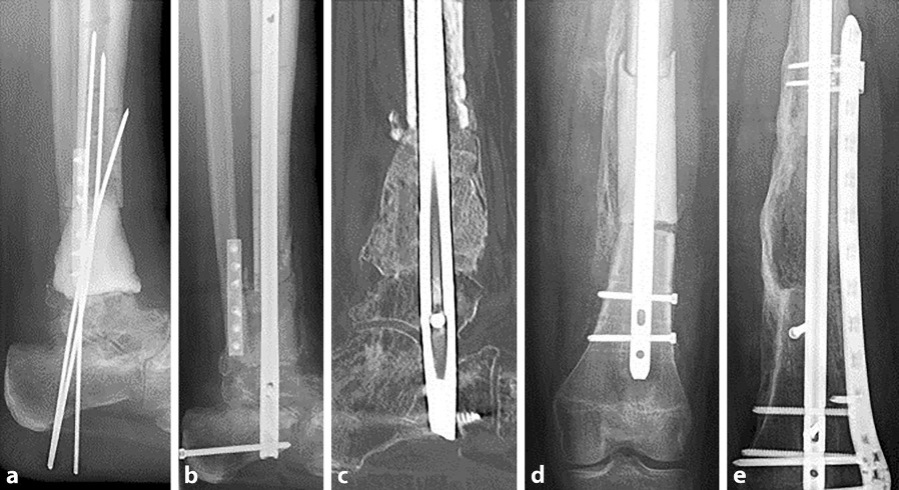
**a** Segmentdefekt der distalen Tibia mit Verlust des oberen Sprunggelenkes, Spacer und temporäre Stabilisierung mit Kirschner-Drähten, **b** MT mit 2 Femurköpfen und mittels RIA gewonnener Spongiosa, retrograde Marknagelarthrodese des Sprunggelenkes, **c** CT nach 4 Monaten mit vollständiger Konsolidierung; **d** 9 cm langer Segmentdefekt am Femurschaft, temporäre Osteosynthese durch Marknagel, **e** MT durch 2 Femurköpfe und RIA-Spongiosa, vollständige Konsolidierung nach 4,5 Monaten, hochstabile Osteosynthese durch Nagel und Plattenosteosynthese

Unabhängig von der Genese des Knochendefektes (Trauma, Infektion, Tumor) wird im ersten Schritt der Technik nach entsprechender Knochenresektion ein Polymethylmethacrylat(PMMA)-Spacer in den Segmentdefekt eingebracht und typischerweise durch eine interne oder externe Osteosynthese stabilisiert. Im eigenen Vorgehen werden auch bei Infektionen fast immer temporäre interne Osteosyntheseverfahren angewendet (Abb. 2a, d).

## Masquelet Schritt 2: Entfernung des Spacers, Spongiosaplastik und definitive Osteosynthese

Im zweiten Schritt der MT wird 6 Wochen nach Einbringen des Spacers bei klinisch fehlenden Zeichen einer Infektion die Masquelet-Plastik durchgeführt. Dabei wird der Spacer unter Schonung der Membran entfernt. Ebenso erfolgt ggf. die Entfernung einer temporären Osteosynthese. Die Resektionsränder des Knochens werden subtil angefrischt, die Vitalität des Knochens wird so überprüft, es erfolgt ein nochmaliges Débridement. Nach Ausmessen der Defektgröße, Kontrolle auf Bluttrockenheit und Spülung wird temporär eine feuchte Tamponade eingelegt.

In Abhängigkeit von Lokalisation und Ausmaß des Knochendefektes werden Varianten der MT und Osteosynthese gewählt. Für ein optimales Ergebnis sollte besonderes Augenmerk auf eine hohe Primärstabilität des Gesamtkonstrukts gelegt werden. Unsere eigenen klinischen Erfahrungen zeigen, dass mit einer lediglich überbrückenden Osteosynthese keine ausreichende Stabilität erzielt werden kann, was nicht selten zu einer pseudarthrotischen Fehlheilung an den Grenzzonen zum originären Knochen führt. Insofern besteht das grundlegende Ziel darin, dem Knochentransplantat neben der Defektauffüllung auch einen größtmöglichen lasttragenden Effekt zukommen zu lassen. Jedoch ist die biologische Verfügbarkeit von autologem Knochenmaterial limitiert. Dies impliziert notwendige Modifikationen der originalen MT, welche im Folgenden beschrieben werden.

### Defektauffüllung mit solidem Knochentransplantat und Beckenkammspongiosa

Defekte bis 5 cm an der unteren und bis zu 10 cm an der oberen Extremität können in der Regel vollständig mit autologem Knochen aufgefüllt werden. Hierfür werden bi- oder trikortikale Beckenkammspäne verwendet, die press-fit in den Segmentdefekt eingebracht werden. Sollte weiteres solides Knochenmaterial benötigt werden, können spongiöse Blöcke aus allogenen Femurköpfen verwendet werden, die vor der Implantation mit zerkleinerter, autologer Beckenkammspongiosa ummantelt werden.

### Defektauffüllung mit soliden thermodesinfizierten dekortizierten Femurköpfen und RIA-Spongiosa-Ummantelung

Besteht der Segmentdefekt an der Tibia, erfolgt die Entnahme der Spongiosa mit dem RIA orthograd aus dem gleichseitigen Femur. Sind am proximalen Femur Implantate vorhanden, gewinnen wir die Spongiosa retrograd. Alternativ können kontralaterales Femur oder Tibia als Entnahmestelle dienen. Um iatrogene Frakturen bei der Entnahme zu vermeiden, sind ein korrekter Eintrittspunkt, ein Ausmessen des Markraumes und die BV-Kontrolle in 2 Ebenen erforderlich. Durch die Kombination mit Spenderknochen sind 10 cm^3^ Spongiosa für die Rekonstruktion eines Segmentdefektes von 4 cm ausreichend. Entsprechend werden etwa 50 ml RIA-Spongiosa für die Rekonstruktion eines 20 cm langen Defektes benötigt.

**Abb. 3:**
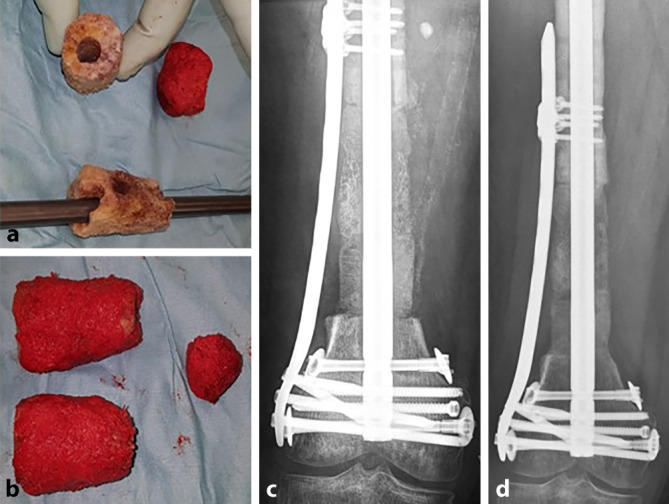
**a** Angepasste thermodesinfizierte Femurköpfe, **b** Ummantelung der Femurköpfe mittels RIA-entnommener Spongiosa; **c** perlschnurartig aufgefädelte Femurköpfe, stabile Osteosynthese durch Marknagel und Platte; **d** schmerzfreie Vollbelastung nach 5 Monaten, weitgehende Konsolidierung

**Abb. 4:**
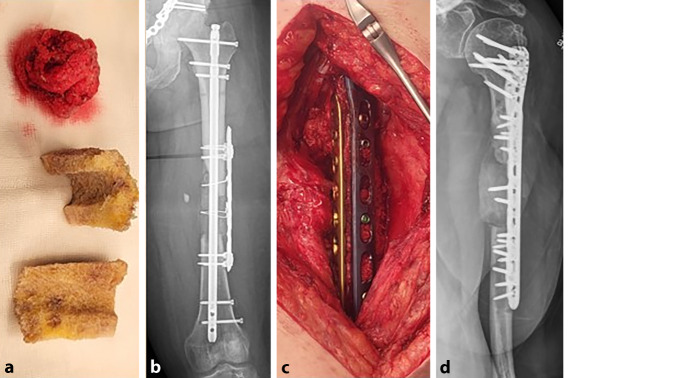
**a** Halbierter dekortizierter Femurkopf und RIA; **b** Sicherung der halbierten Femurköpfe durch Cerclagen; **c**,**d** MT durch einen angepassten thermodesinfizierten Femurkopf und RIA am Humerus, winkelstabile Doppelplattenosteosynthese mit Fixierung des Femurkopfes durch 2 Kopfverriegelungsschrauben

Die Rekonstruktion ausgedehnter dia-/metaphysärer Defekte von mehr als 10 cm macht die Verwendung von soliden Transplantaten unumgänglich. Als allogenes Trägermaterial bieten sich thermodesinfizierte Femurköpfe an. Sie sind in unbegrenzter Menge kommerziell erhältlich und gegenüber synthetisch hergestellten Scaffolds deutlich kostengünstiger. Die Anzahl der verwendeten Spenderfemurköpfe orientiert sich an der Ausdehnung des Segmentdefektes. Abhängig davon, ob bei der Entnahme der Femurköpfe Anteile des Schenkelhalses entfernt oder erhalten wurden, können spongiöse Zylinder zwischen 3 und 6 cm hergestellt werden. Für einen 10 cm langen Defekt werden also 2 bis 3 Femurköpfe benötigt. Erfolgt die Osteosynthese mit einem Marknagel, werden die Femurköpfe kanüliert und 1 mm über den Nageldurchmesser aufgebohrt. Es erfolgt die vollständige Entfernung der Kortikalis, da nach unserer Erfahrung eine Revitalisierung des Spenderknochens mit Einwachsen von Blutgefäßen effektiv nur bei Vorhandensein einer spongiösen Oberfläche des Spenderknochens stattfindet. Danach werden die Köpfe dem Durchmesser des Defektes angeglichen. Im nächsten Schritt erfolgt *ex situ* die Ummantelung der Knochenzylinder mit der RIA-Spongiosa (Abb. 3a, b). Der Marknagel wird anschließend bis zur Defektzone eingeschlagen, die Femurköpfe werden perlschnurartig auf den Nagel aufgefädelt. Dabei muss ausreichend RIA-Spongiosa an die Kontaktflächen der Femurköpfe und der Resektionsränder des Knochens angelagert werden. Nach Möglichkeit werden die Köpfe press-fit eingebracht bzw. Kompression auf die Masquelet-Plastik aufgebracht. Kleinere Defekte werden mit verbleibender RIA aufgefüllt. Wurde bereits in einem vorangegangenen Eingriff eine Marknagelosteosynthese durchgeführt, werden die Femurköpfe halbiert und um den liegenden Nagel angebracht. Die Sicherung erfolgt durch Cerclagen oder eine additive winkelstabile Platte. Erfolgt die Osteosynthese mit winkelstabilen Platten, werden die Köpfe *en bloc* implantiert und mit Kopfverriegelungsschrauben an der Platte fixiert (Abb. 4a, b).

Absolute Stabilität von Osteosynthese und Masquelet-Plastik sind die Voraussetzungen einer erfolgreichen Therapie. Liegen Defekte im Isthmusbereich langer Röhrenknochen vor, kann die Osteosynthese mit einem ausreichend starken Nagel genügen. Liegt keine ausreichende Stabilität vor, sollten zusätzlich winkelstabile Platten verwendet werden (Abb. 2e, 3d und 4b). Im Gelenkbereich werden typischerweise winkelstabile Doppelplattenosteosynthesen angewandt (Abb. 1d, e).

Von bisher 34 mit dieser Modifikation der MT behandelten Patienten musste bei lediglich 3 Patienten aufgrund einer Rezidivinfektion die Masquelet-Plastik ganz oder teilweise entfernt werden. Nach Sanierung der Rezidivinfektion wurde bei diesen Patienten erneut eine MT durchgeführt. In allen anderen Fällen kam es zur Konsolidierung; Revisionseingriffe mussten nicht durchgeführt werden. Die Länge des Knochendefektes hat dabei keinen wesentlichen Einfluss auf die Konsolidierungsrate und die Zeit bis zum Erreichen der Vollbelastung.

## Nachbehandlung

Die dargestellten Varianten verbinden stabile interne Osteosynthesen mit einer stabilen Spongiosaplastik. Hierdurch ist in jedem Fall mindestens eine postoperative Übungsstabilität gegeben. Bei Defekten der unteren Extremitäten ist in der Regel unmittelbar postoperativ eine Teilbelastung mit 20 kg möglich. Hierdurch können im Gegensatz zu anderen Methoden der Defektrekonstruktion langfristige funktionelle Einschränkungen minimiert werden.

Nach 6 Wochen erfolgt eine schrittweise Aufbelastung; die Vollbelastung sollte 12 Wochen postoperativ erreicht sein. Abweichungen vom Standard können in Abhängigkeit von den radiologischen Kontrollen oder individueller Faktoren, wie der Fähigkeit zur Teilbelastung oder dem Körpergewicht, sinnvoll sein. Typischerweise führen wir Röntgenkontrollen nach 6 und 12 Wochen sowie nach 6 und 12 Monaten durch. Neben der Beurteilung des Konsolidierungsfortschritts ist dabei die frühzeitige Erkennung möglicher Implantatkomplikationen wichtig.

## Zusammenfassung

Die MT ist ein zweizeitiges Verfahren zur Rekonstruktion großer Knochendefekte. Durch vielfältige Modifikationen der Originaltechnik können anhand der Literatur aktuell keine allgemeingültigen Aussagen zur Wertigkeit der MT getroffen werden. Mit den hier vorgestellten Modifikationsvarianten können grundsätzlich sehr gute Konsolidierungsergebnisse bei zugleich geringer Komplikationsrate erzielt werden.

Hierbei erfüllen beide Varianten die von Giannoudis et al. beschriebenen Eckpfeiler des Diamond-Konzeptes für eine erfolgreiche biologische Rekonstruktion von knöchernen Defekten. Sie verbinden eine hohe Stabilität von Osteosynthese und Spongiosaplastik mit den Faktoren Osteokonduktion (Scaffold), Osteoinduktion (Wachstumsfaktoren) und Osteogenese (Zellen).

Durch primäre Übungsstabilität und Teilbelastung sowie die zeitgerechte Konsolidierung und Vollbelastung sind die Ergebnisse mit denen einer primären Frakturversorgung vergleichbar.

Weitere Studien zur MT sollten auf die Analyse der wichtigsten Zielgrößen Konsolidierung, Infekteradikation sowie Behandlungszeit und funktionelles Outcome abzielen.
